# Establishing C-X-C motif chemokine receptor 4 as a novel imaging target in giant cell arteritis

**DOI:** 10.1186/s13075-026-03747-4

**Published:** 2026-02-11

**Authors:** Matthias Fröhlich, Sebastian E. Serfling, Michael Gernert, Konstanze Guggenberger, Takahiro Higuchi, Elena Gerhard-Hartmann, Alexander Weich, Samuel Samnick, Marc Schmalzing, Thorsten A. Bley, Andreas K. Buck, Rudolf A. Werner

**Affiliations:** 1 Department of Internal Medicine II, Rheumatology/Clinical Immunology, University Hospital Würzburg, Würzburg, 97080 Germany; 2 Department of Nuclear Medicine, University Hospital Würzburg, Würzburg, Germany; 3 Department of Diagnostic and Interventional Radiology, University Hospital Würzburg, Würzburg, Germany; 4 Faculty of Medicine, Dentistry and Pharmaceutical Sciences, Okayama University, Okayama, Japan; 5 Department of Pathology and Comprehensive Cancer Center Mainfranken, Julius- Maximilian University of Würzburg, Würzburg, Germany; 6 Department of Internal Medicine II, Gastroenterology, University Hospital Würzburg, Würzburg, 97080 Germany; 7 European Neuroendocrine Tumor Society (ENETS) Center of Excellence, NET Zentrum, University Hospital Würzburg, Würzburg, 97080 Germany; 8 The Russell H Morgan Department of Radiology and Radiological Science, Division of Nuclear Medicine and Molecular Imaging, Johns Hopkins School of Medicine, Baltimore, MD U.S.; 9 Department of Nuclear Medicine, Clinical Center for Nuclear Medicine at Ludwig Maximilian University Munich, Munich, 81377 Germany

## Abstract

**Background:**

PET imaging in giant cell arteritis (GCA) is crucial for diagnosis. New tracers such as C-X-C motif chemokine receptor 4 (CXCR4) enable to directly visualize inflammatory cells as they are expressed on leukocytes. We aimed to test the value of CXCR4-targeted PET in GCA.

**Methods:**

Ten treatment-naïve patients with confirmed large-vessel GCA underwent both [^18^F]FDG and [^68^Ga]PentixaFor PET/CT scans within a median of two days, without any therapy in between. Thirteen arterial segments per patient were analyzed. Visual interpretation and quantitative target-to-background ratios (TBR; arterial SUVmax divided by superior vena cava SUVmean) were calculated, including per-patient mean TBRs. Five patients without clinical or diagnostic evidence of vasculitis served as Non-GCA controls. Flow cytometry was used to quantify CXCR4 expression on leukocyte subsets, reported as normalized median fluorescence intensity (NMFI).

**Results:**

All GCA patients showed positive scan findings on both [^18^F]FDG and [^68^Ga]PentixaFor PET/CT. Mean vascular TBRs were 2.43 ± 0.90 for FDG and 1.76 ± 0.76 for PentixaFor (*P* = 0.07), indicating similar large-vessel uptake. Segment-level analysis showed no significant differences in 10/13 vascular regions, although FDG uptake was higher in selected arteries. PentixaFor TBR was significantly lower in Non-GCA controls (1.15 ± 0.10 vs. 1.76 ± 0.76; *P* = 0.01), supporting its specificity for inflammation. Blood pool SUVmean did not differ, suggesting minimal signal spill-in. CXCR4 expression was highest on naïve T-helper cells and monocytes.

**Conclusions:**

CXCR4-targeted [^68^Ga]PentixaFor PET/CT provides an imaging pattern comparable to [^18^F]FDG PET/CT in untreated GCA and reliably differentiates between inflamed and non-inflamed vessels. These findings support CXCR4 PET as a promising, mechanistically grounded imaging approach that merits further evaluation in larger patient cohorts.

**Clinical trial number:**

ClinicalTrials.gov NCT05604482. Registered 3 November 2022.

**Supplementary Information:**

The online version contains supplementary material available at 10.1186/s13075-026-03747-4.

## Introduction

Affecting cranial and large vessels, giant cell arteritis (GCA) can pose a diagnostic challenge for the treating rheumatologist [1, 2], in particular in the absence of specific symptoms, such as headache [3]. Thus, for establishing diagnosis and to determine disease extent, [^18^F]-2-fluoro-2-deoxy-D-glucose ([^18^F]FDG) PET/CT has been applied in indeterminate cases, which is also endorsed by the recent EULAR recommendations for imaging in GCA and the new joint classification criteria of the American College of Rheumatology (ACR) and European League Against Rheumatism (EULAR) [4, 5]. Nonetheless, [^18^F]FDG may have some inherent limitations in GCA as it visualizes glycolytic activity of various activated immune and stromal cell populations, which limits its ability to attribute the PET signal to specific leukocyte subsets involved in the immunopathology of GCA [6]. Moreover, the use of glucocorticoids triggers glycolysis pathway inhibition, thereby limiting precise scan interpretation [6]. GCA is a T-cell-driven disease in which CD4 + T cells and CD8 + T cells play a critical role in the pathophysiology of the inflammatory process in the vessel wall [7, 8]. Among others, chemokines have been advocated to be crucially involved in CD8 + T-cell adhesion, migration, and attraction of other immune cells to infiltrate vessel walls [7], rendering chemokine receptors as attractive targets for molecular imaging in GCA. For instance, the C-X-C motif chemokine receptor 4 (CXCR4)-targeting PET agent [^68^Ga]PentixaFor has been applied to varying other immune-related diseases, such as severe acute respiratory syndrome coronavirus type 2, myocardial infarction or systemic lupus erythematosus [9–12]. Of note, beyond favorable read-out capabilities with high image contrast, this chemokine receptor-specific radiotracer also provided prognostic potential to determine patients at increased risk for clinical deterioration [10].

In the present Phase II trial, we aimed to investigate whether CXCR4 could be a useful target in GCA by analyzing the CXCR4 expression on different leukocyte subsets. We also aimed to determine the diagnostic performance of chemokine-receptor imaging using [^68^Ga]PentixaFor in treatment-naïve GCA patients, thereby investigating its potential as a non-invasive inflammatory-directed biomarker.

## Material and methods

### Patients

#### General

Consecutive patients with large vessel (LV) GCA were prospectively recruited at the University Hospital of Würzburg. The final diagnosis of GCA was established by board-certified rheumatologists based on clinical evaluation in accordance with current EULAR and ACR guidelines, integrating patient history, laboratory results, and imaging findings [5, 13]. As part of routine work-up, an [^18^F]FDG PET/CT was conducted, while an additional [^68^Ga]PentixaFor PET/CT was performed within 2 days (median) in 10 patients (time interval between scans, 2.5 ± 1.4 days). Only patients with clinically suspected GCA and positive [^18^F]FDG PET/CT findings were enrolled for [^68^Ga]PentixaFor PET/CT imaging; patients with negative [^18^F]FDG PET/CT were not included, in line with ethical approval and study design. All subjects were therapy-naïve at time of imaging and remained without treatment between scans. For more information on the eligibility criteria, see NCT05604482. The study was approved by the local ethics committee (University of Wuerzburg, 30/21-me), and written informed consent was obtained from all participants. Patients’ characteristics are shown in Table 1. In addition, as part of an approved retrospective cohort of patients scheduled for CXCR4-directed molecular imaging (University of Wuerzburg, 20210726 02), individuals diagnosed with neuroendocrine tumors and imaged with [^68^Ga]PentixaFor PET/CT served as comparative controls. Those individuals had no clinical or diagnostic evidence of GCA or any other inflammatory disease (Non-GCA group).

**Table 1:** Patients’ characteristics. Percentages are given in parentheses. Metric parameters are given in mean ± standard deviation. CRP = C-reactive protein

Parameter	
Age (years) (mean ± SD)	66.9 ± 7.0
Female	6/10 (60)
Blood values at time of imaging	
CRP (mg/dl)	6.36 ± 4.09
Erythrocyte Sedimentation Rate (mm/1st hour)	81.88 ± 15.38
Leucocytes (x10^9^/L)	9.98 ± 4.04
Symptoms at time of imaging	
Headache	3/10 (30)
Temporal artery abnormalities	0/10 (0)
Occipital/Neck pain	1/10 (10)
Visual symptoms	1/10 (10)
Other ischemic symptoms	1/10 (10)
Joint-associated symptoms	3/10 (30)
Weight loss	7/10 (70)
Night sweats	8/10 (80)
General weakness	8/10 (80)
Fever	1/10 (10)
Relapse	
No. relapses	3/10 (30)
Follow-up period (weeks)	53.4 ± 30.1
Time to first relapse (weeks)	29.9 ± 16.8

### Imaging

#### PET/CT acquisition

[^18^F]FDG was synthesized in-house with a 16 MeV Cyclotron (Würzburg; GE PET trace 6; GE Healthcare, Milwaukee, WI, USA). [^68^Ga]PentixaFor was produced using a dedicated synthesis module (Scintomcis) along with single-use cassette kits (ABX, Radeberg, Germany) [14]. Scans were performed on a PET/CT scanner (Siemens Biograph mCT 64 or mCT 128, Siemens, Knoxville, TN, USA). For [^18^F]FDG, patients fasted at least 6 h prior to injection of 219.8 ± 42.6 MBq. Scans were acquired after 60 min post-injection, using non-contrast-enhanced CT as described in [15]. For [^68^Ga]PentixaFor, fasting was not mandatory and scans were conducted 60 min after the administration of 143.7 ± 13.1 MBq. Acquisition parameters of respective low-dose CTs are provided in [16]. PET/CT data were reconstructed according to standard protocols and quality controls for hybrid imaging components were conducted on a regular basis [15].

#### PET/CT analysis

PET/CT images were evaluated in a consensus read by two experienced nuclear medicine physicians (S.E.S., R.A.W.) using a dedicated workstation (Syngo.Via; V10B; Siemens Healthcare, Erlangen, Germany); interobserver variability was not assessed formally in this pilot cohort. PET, CT, and hybrid PET/CT image overlays were analyzed in all patients. On a visual inspection, both readers decided on a positive or negative overall scan impression [17]. Quantitative evaluation (target-to-background ratios [TBR]) were primarily conducted [18, 19]. To provide a reference standard for evaluating [^68^Ga]PentixaFor uptake in vessels of GCA patients, subjects of the Non-GCA group were analyzed accordingly. In total, 13 arterial segments were assessed: ascending aorta, aortic arch, descending thoracic aorta, abdominal aorta, pulmonary artery, both carotid, subclavian, axillary, and vertebral arteries [20]. By placing volumes of interest (VOIs), maximum standardized uptake values (SUV_max_) from all vessels were recorded. In addition, VOIs were drawn on the vena cava superior (VCS, to determine mean SUV [SUV_mean_]). Target-to-background ratios (TBR) were then calculated by dividing SUV_max_ of every artery by SUV_mean_ of VCS. An average TBR among all investigated vessels was also provided [20]. For each patient, an average TBR was calculated by taking the mean of the 13 segment-wise TBR values; these per-patient averages were then summarised across the cohort.

### Immunophenotyping of leukocytes

Peripheral blood was obtained from all patients on the day of [^68^Ga]PentixaFor PET/CT. C-reactive protein (CRP) and white blood cell count (WBC) were determined in all subjects, while erythrocyte sedimentation rate (ESR) was available in *n* = 8/10 (80%). Immunophenotyping of leukocytes was done by flow cytometry using a Navios cytometer (Beckman Coulter, Krefeld, Germany). Preparation of peripheral blood samples including staining, washing and erythrocyte lysing is described elsewhere [21]. Six panels were stained in parallel: Myeloid, B cell, and T cell panels, each with CXCR4-PE or IgG2a-PE isotype control. The normalized median fluorescence intensity (NMFI) on each leukocyte population was calculated by dividing the median fluorescence intensity (MFI) after staining with CXCR4 by the corresponding MFI of the isotype control. Antibodies used are shown in Supplementary Table S1 and definition of leukocyte subpopulations are shown in Supplementary Table S2. Gating strategies for all cell types are shown in Supplementary Figure S1.

### Statistics

For statistical analysis, Prism (version 10.0.1, GraphPad, San Diego, CA, USA) and R (version 4.3.1, R Core Team, 2023) were used. Continuous variables are presented as mean ± standard deviation. Group-wise comparisons of TBR values between [^18^F]FDG and [^68^Ga]PentixaFor, and between GCA and Non-GCA, were performed using paired Wilcoxon signed-rank tests because of the small sample size. Interquartile ranges (IQR) were additionally calculated as descriptive measures of dispersion, but no inferential testing was based on IQR. Correlations were analysed using Spearman’s rank correlation. A two-sided *P* < 0.05 was considered statistically significant.

## Results

### [^68^Ga]PentixaFor-derived TBR revealed comparable findings in arterial segments between both radiotracers

**Fig. 1:**
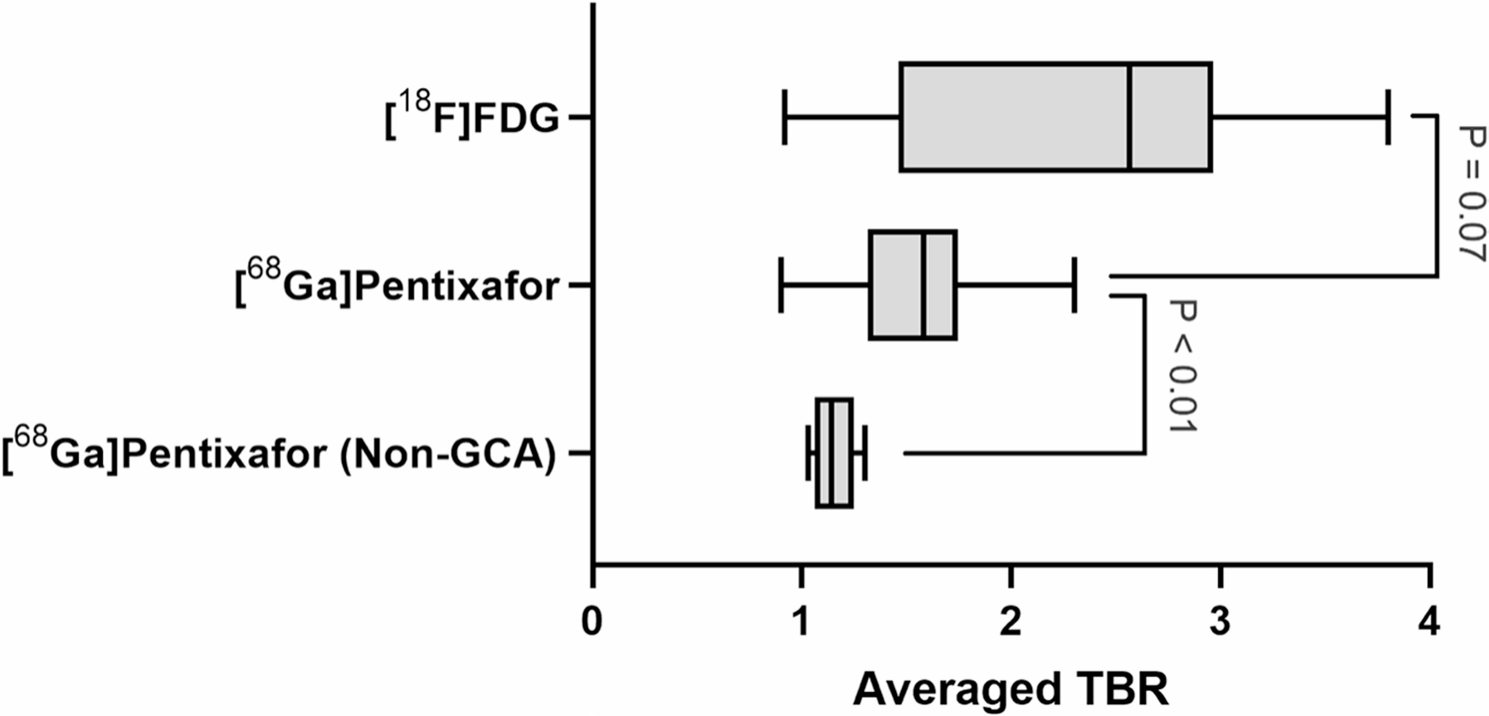
Averaged target-to-background ratio (TBR) of [^18^F]FDG and [^68^Ga]PentixaFor PET in giant cell arteritis (GCA) and non-GCA patients serving as controls. While TBR of [^18^F]FDG and [^68^Ga]PentixaFor in GCA was comparable, the uptake was significantly increased in the latter group when compared to Non-GCA subjects imaged with [^68^Ga]PentixaFor

Overall scan impression was positive in all 10/10 (100%) GCA patients for both [^18^F]FDG and [^68^Ga]PentixaFor PET/CT. For each patient, a per-patient average TBR was calculated as the mean of the 13 segment-wise TBR values. The average TBR across all patients was 2.43 ± 0.90 for [^18^F]FDG and 1.76 ± 0.76 for [^68^Ga]PentixaFor, with no statistically significant difference between the two tracers (*P* = 0.07; Fig. 1). On a per-vessel analysis, [^18^F]FDG-derived TBR was significantly higher in 3/13 (23.1%) arterial segments (right subclavian, ascending and descending aorta; *P* ≤ 0.04), while in the remaining 10/13 (76.9%) territories, no significant differences in TBR were observed (*P* ≥ 0.07). These findings indicate that, in the majority of vascular territories, CXCR4-directed uptake parallels [^18^F]FDG-defined inflammation in treatment-naïve GCA.

In an exploratory analysis, [^18^F]FDG- and [^68^Ga]PentixaFor-derived TBR values showed moderate-to-high correlations across most arterial territories (Pearson *r* = 0.20–0.83, highest in the ascending aorta), suggesting that segments with higher [^18^F]FDG uptake generally also display higher CXCR4-directed uptake (Supplementary Table S3). Given the small sample size, these correlation data are considered descriptive and were not used to derive diagnostic cut-offs.

### [^68^Ga]PentixaFor TBR in Non-GCA controls

To assess tracer uptake in non-inflamed vessels, [^68^Ga]PentixaFor PET/CT scans from five patients without clinical or diagnostic evidence of GCA or other inflammatory disease were analysed as a Non-GCA control group (*N* = 5). The average vascular TBR was significantly lower in Non-GCA subjects compared with GCA patients (1.15 ± 0.10 vs. 1.76 ± 0.76; *P* = 0.01; Fig. 1). This finding supports the interpretation that increased [^68^Ga]PentixaFor uptake in GCA reflects active vascular inflammation rather than constitutive background signal.

### Comparable aortic blood pool activity between [^18^F]FDG and [^68^Ga]PentixaFor

To evaluate potential spill-in from CXCR4-positive leukocytes in the blood pool, SUVmean values were measured in the ascending and abdominal aorta. No significant differences were observed between [^18^F]FDG and [^68^Ga]PentixaFor in these regions (*P* = 0.743 and *P* = 0.690, respectively; Supplementary Figure S2), arguing against substantial contamination of the vessel wall signal by circulating leukocytes.

**Fig. 2:**
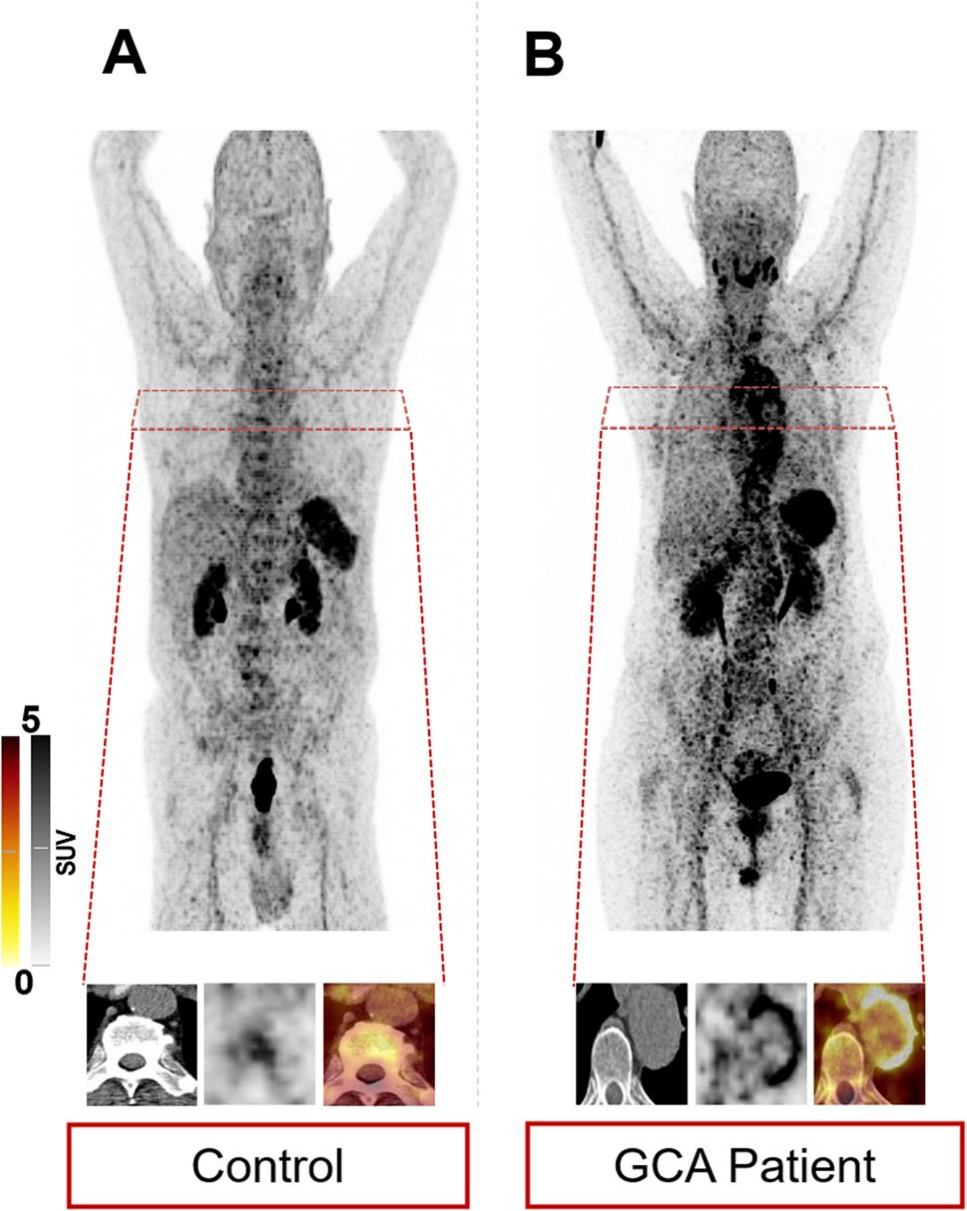
Maximum intensity projections and trans-axial head-to-head comparisons of the thoracic aorta after injection of [^68^Ga]PentixaFor. **A** Non-GCA control subject with almost no discernible tracer uptake in the vessel wall. **B** GCA patient with pronounced circumferential vessel wall uptake. These examples illustrate the range of CXCR4-directed vascular signal from non-inflamed to highly inflamed arteries [18]

Figure 2 shows two GCA subjects with low and high increased vessel wall uptake, along with an individual from the non-GCA group with almost no discernible radiotracer accumulation in the vessels.

### Flow cytometry revealed broad CXCR4 expression on leukocyte populations, with highest upregulation on naïve T-helper-cells and monocytes

**Fig. 3:**
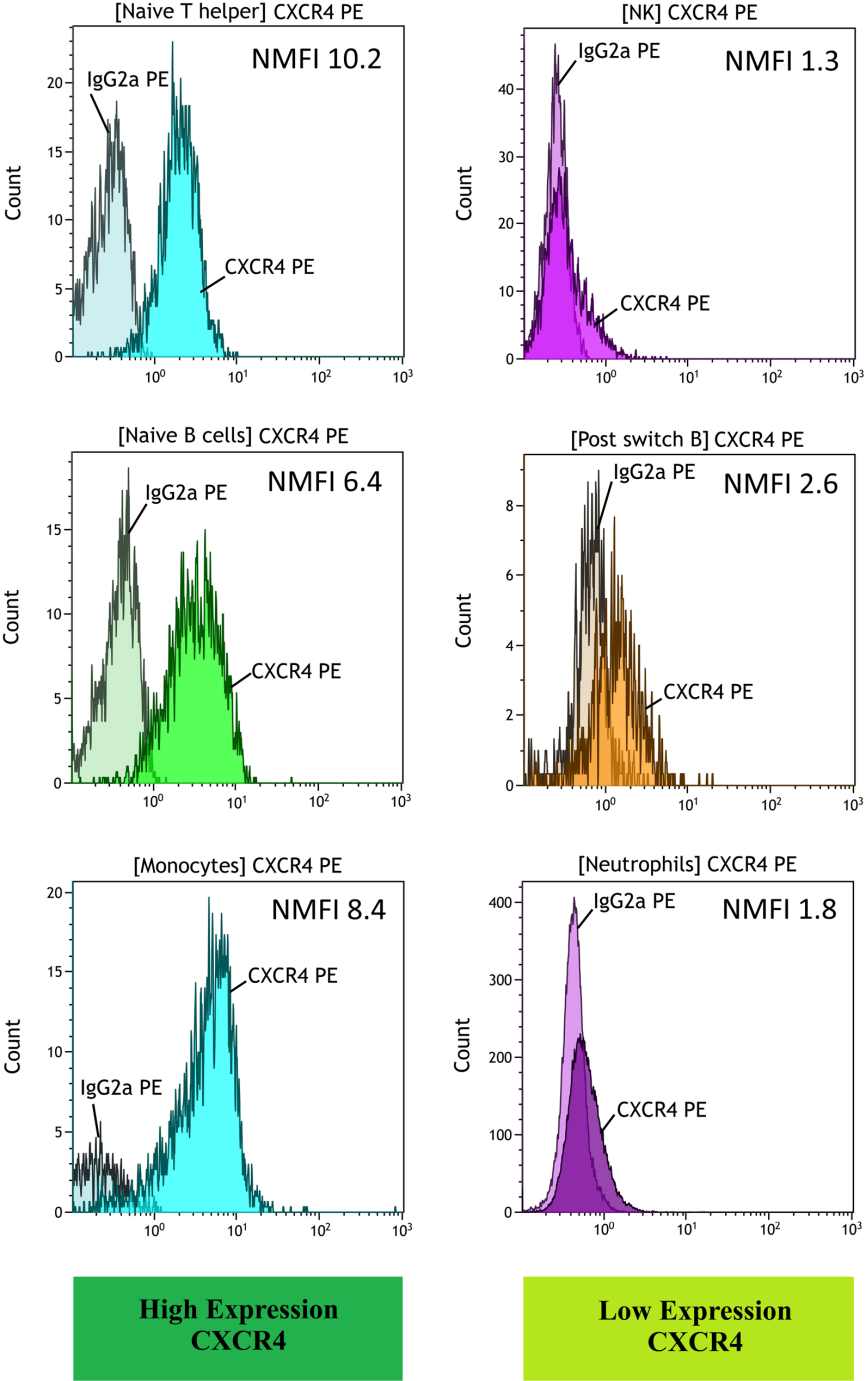
Flow cytometry of peripheral-blood leucocytes. CXCR4 expression shown as normalized median fluorescence intensity (NMFI; CXCR4 staining compared to isotype control [IgG2a]) in the T cell panel (upper row) in the B cell panel (middle row) and in the myeloid panel (lower row). The left column shows the respective subpopulation of each panel with the highest CXCR4 expression and the right column shows the population with the lowest subpopulation

Flow cytometry showed broad expression of CXCR4 on leukocyte populations. Within the lymphocytes, naïve cell populations showed a higher CXCR4-expression compared to memory subsets. The highest numerical CXCR4-NMFI was detected on naïve T-helper cells (mean NMFI 10.19 ± 6.06), and for B-cells, again, naïve B-cells (6.37 ± 1.65). In contrast, post-switch memory B-cells or exhausted B cells (represented by CD21low B-cells) exhibited lower CXCR4-expressions (2.64 ± 1.08 and 2.22 ± 1.51, respectively). Within the myeloid cells, monocytes expressed the highest CXCR4-NMFI (8.35 ± 13.60). The corresponding flow cytometry plots are shown in Fig. 3.

## Discussion

In this phase II trial, flow cytometry revealed highest CXCR4 expression on naïve T-helper cells and monocytes among circulating leukocytes in GCA patients, providing a biologically plausible context for the vascular [^68^Ga]PentixaFor uptake observed on PET/CT. Together, these in vivo and ex vivo data suggest that CXCR4 is involved in the inflammatory response in GCA, but they do not allow us to define the exact cellular contributors to the PET signal in the vessel wall. As such, the herein presented findings open avenues for further research, e.g., to establish CXCR4-targeted imaging for therapeutic guidance of novel anti-inflammatory drugs or identification of high-risks prone to relapse. Finally, the retrospective inclusion of a control cohort without evidence of GCA or other inflammatory conditions allowed us to establish a reference for [^68^Ga]PentixaFor uptake in non-inflamed vessels, supporting the specificity of CXCR4-targeted PET for vascular inflammation.

Focusing on treatment-naïve GCA patients, we aimed to establish CXCR4 as a more tissue-specific, inflammatory-driven target in GCA. In a flow cytometry analysis of treatment-naïve individuals, we revealed a plausible profile of a broad upregulation of leukocytes, in particular on monocytes and naïve T-helper-cells. In this regard, higher rates of naïve cell populations are a hallmark of inflammation triggering an increased white blood cell count in the peripheral blood [22–25]. In contrast to mature white blood cells, however, those naïve cell types have not yet had contact with a specific antigen, which leads to further differentiation including migration into the target tissue, such as affected vessels [24, 26]. Thus, the herein presented results from blood profiling provide a rationale for the observed increased [^68^Ga]PentixaFor uptake in the arterial wall in untreated GCA patients. Although CXCR4 is prominently expressed on circulating leukocytes, which could theoretically increase tracer retention in the blood pool, our quantitative analysis in two major vascular regions revealed no significant SUV_mean_ differences between [^18^F]FDG and [^68^Ga]PentixaFor. This finding argues against substantial spill-in effects from unspecific blood pool activity and supports the reliability of CXCR4-targeted PET signal as reflective of true vessel wall involvement under the applied imaging conditions.

In GCA, novel serum biological biomarkers to determine disease extent have been extensively investigated, including procalcitonin, interleukin or interferon-γ, but have been advocated to provide limited value in active disease [27]. This, however, is in contrast to a recent observation on multiplex chemokine plasma-based beads assays, including varying CXC ligands [28], which may even have the potential to predict relapse. Those approaches, however, only deal with components attributable to active GCA, which are shed in the patient’s bloodstream, while neglecting intrinsic disease multifariousness or disease extent throughout the patient’s body. This is in contrast to a PET-based radioactive biomarker assay, which provides an in-depth read-out of the current target expression of the entire vasculature [29]. To date, [^18^F]FDG serves as the reference PET agent in GCA, in particular when extracranial vessels are affected, but required fasting, physiological uptake or radiotracer accumulation in tissue with increased glycolytic activity limit a broader application [20]. As an image biomarker of chemotaxis, the CXCR4-directed PET probe [^68^Ga]PentixaFor, however, may overcome those issues, as it enables for a direct visualization of the CXCR4 expression on CD45 + leukocytes and T-cells [30, 31]. However, [^18^F]FDG PET/CT does not provide a direct read-out of distinct leukocyte subsets but rather reflects increased glucose metabolism in activated immune and vascular wall cells, including macrophages, T cells and other inflammatory populations. In contrast, CXCR4-directed PET visualises a chemokine receptor that is broadly expressed on circulating leukocytes, with highest expression in naïve T-helper cells and monocytes in our cohort. While this pattern is biologically plausible, our data do not allow us to assign the vascular CXCR4 signal to a single cell type [32]. Nonetheless, the herein investigated head-to-head comparison between [^18^F]FDG and [^68^Ga]PentixaFor revealed comparable findings on a visual and quantitative level, except of less scatter for the latter imaging agent. As such, despite its more target-specific properties on a (sub)cellular level, [^68^Ga]PentixaFor imaging may suffer from varying physicochemical drawbacks, including the limited resolution of Gallium labeling relative to fluorine radiochemistry [33]. As such, ^18^F-labeled CXCR4-directed radiotracers should be subject to future studies in patients with GCA [34], which may then provide improved read-out capabilities relative to the more unspecific PET probe [^18^F]FDG. We deliberately refrained from defining positive versus negative vascular segments based on TBR cut-offs, as no validated [^18^F]FDG threshold for large-vessel vasculitis exists to date. Jointly optimising cut-offs for both [^18^F]FDG and [^68^Ga]PentixaFor TBR in a 10-patient cohort would constitute a two-dimensional optimisation problem with several arbitrary decisions and limited clinical interpretability. Instead, we provide per-segment correlations between both tracers as an exploratory indicator that higher [^18^F]FDG uptake is generally paralleled by higher CXCR4-directed uptake. These correlations may guide future, adequately powered studies that are specifically designed to establish robust, tracer-specific cut-offs.

In addition to mean TBR values, we explored the interquartile range of TBR as a descriptive measure of dispersion and observed numerically lower spread for [^68^Ga]PentixaFor in several vascular territories. Given the small sample size and the post-hoc nature of this analysis, we report these IQR findings qualitatively only and do not base any formal statistical inference on them. They should therefore be interpreted as hypothesis-generating with respect to a potentially more homogeneous CXCR4-directed signal. Nonetheless, beyond imaging, our results on establishing CXCR4 as a potential target in GCA may also have further therapeutic implications. For instance, mavorixafor is an orally available antagonist of CXCR4 [35] and has been tested in clinical trials for various diseases, including primary immunodeficiencies, melanoma and renal cell carcinoma [36, 37]. In an oncological setting, this chemokine receptor-interacting drug has been shown to enhance the immune response by modulating the transport of immune cells and reducing the recruitment of immunosuppressive cells into the tumor microenvironment [38]. In respect to GCA, selective blockage of CXCR4 potentially disrupting disease progression is also currently investigated [39]. In this regard, chemokine receptor-directed imaging then provides a non-invasive read-out of the current CXCR4 expression in the target region (vessels) and in distant organs (liver, myocardium), thereby potentially predicting potential off-target effects of CXCR4 antagonists [40]. Moreover, as recently demonstrated in myocardial infarction, CXCR4-targeted PET could also be used to implement image-piloted therapeutic strategies, e.g., by initiating chemokine receptor-disrupting drugs at the maximum of the PET-based target expression to enhance therapeutic efficacy [9]. Further studies may also shed light on the systemic immune response in active GCA, as CXCR4 PET also allows for visualizing organs involved in hematopoietic activation, including the spleen or bone marrow [30]. Although histopathologic correlation was not feasible in this study, the close temporal proximity of the scans, the treatment-naïve status of all patients, and the consistent clinical signs of active disease support the interpretation that CXCR4 uptake reflects vascular inflammation.

A limitation of this study is the lack of histopathologic confirmation for PET-positive vascular segments, which prevents direct validation of [^68^Ga]PentixaFor uptake as a marker of inflammation. Moreover, the applicability of [^68^Ga]PentixaFor for cranial GCA remains limited due to both technical and ethical constraints. Owing to the small caliber of cranial arteries (e.g., temporal, maxillary) and the spatial resolution limits of Gallium-68 based imaging, reliable assessment of these vessels is technically challenging. In addition, for ethical reasons, patients with suspected isolated cranial GCA could not be included, as delaying treatment to allow sequential imaging is not feasible given the high risk of ischemic complications [13].

An additional methodological limitation concerns a visual, PETVAS-derived (PET vasculitis activity score) evaluation. PETVAS was originally developed and validated for [^18^F]FDG using the liver as reference tissue. Adapting this score to CXCR4 PET is not validated and therefore was not used. We focused on TBR-based quantification, which can be applied consistently to both tracers and showed a clear difference between inflamed and non-inflamed vessels. Furthermore, because image interpretation was performed in a consensus read by two readers, we could not assess interobserver variability for [^68^Ga]PentixaFor-based vascular scoring in GCA; this will need to be addressed in larger validation cohorts. Owing to ethical considerations, CXCR4 PET/CT was only approved in patients with confirmed [^18^F]FDG PET/CT-positive GCA, and not in an unselected population. As such, the present study does not allow assessment of diagnostic accuracy.

## Conclusion

In this phase II trial, flow cytometry revealed increased CXCR4 expression on naïve T-helper-cells and monocytes, thereby rendering this receptor as a potential interventional target in GCA. In addition, CXCR4-directed molecular imaging provides a non-invasive whole-body read-out of the receptor expression in the vasculature and thus, chemokine receptor-directed PET probes may emerge as novel, more target-specific biomarkers in active disease, e.g., for therapeutic guidance of novel anti-inflammatory drugs or identification of high-risks prone to relapse.

## Supplementary Information

Supplementary Material 1.

## Data Availability

Data are not available in accordance to the European regulations regarding data protection and therefore, cannot be provided online or via airmail. However, data is available for on-site revision.
