# Supplementary material for: Establishing C-X-C motif chemokine receptor 4 as a novel imaging target in giant cell arteritis
Source: Arthritis Res Ther. 2026 Feb 11;28:61. doi: 10.1186/s13075-026-03747-4 (PMC12937570; doi:10.1186/s13075-026-03747-4)
Supplement: Supplementary file 1 — Supplementary Material 1. [file 13075_2026_3747_MOESM1_ESM.docx]

**Establishing C-X-C Motif Chemokine Receptor 4 as a Novel Imaging Target in Giant Cell Arteritis**

**Supplement**

**
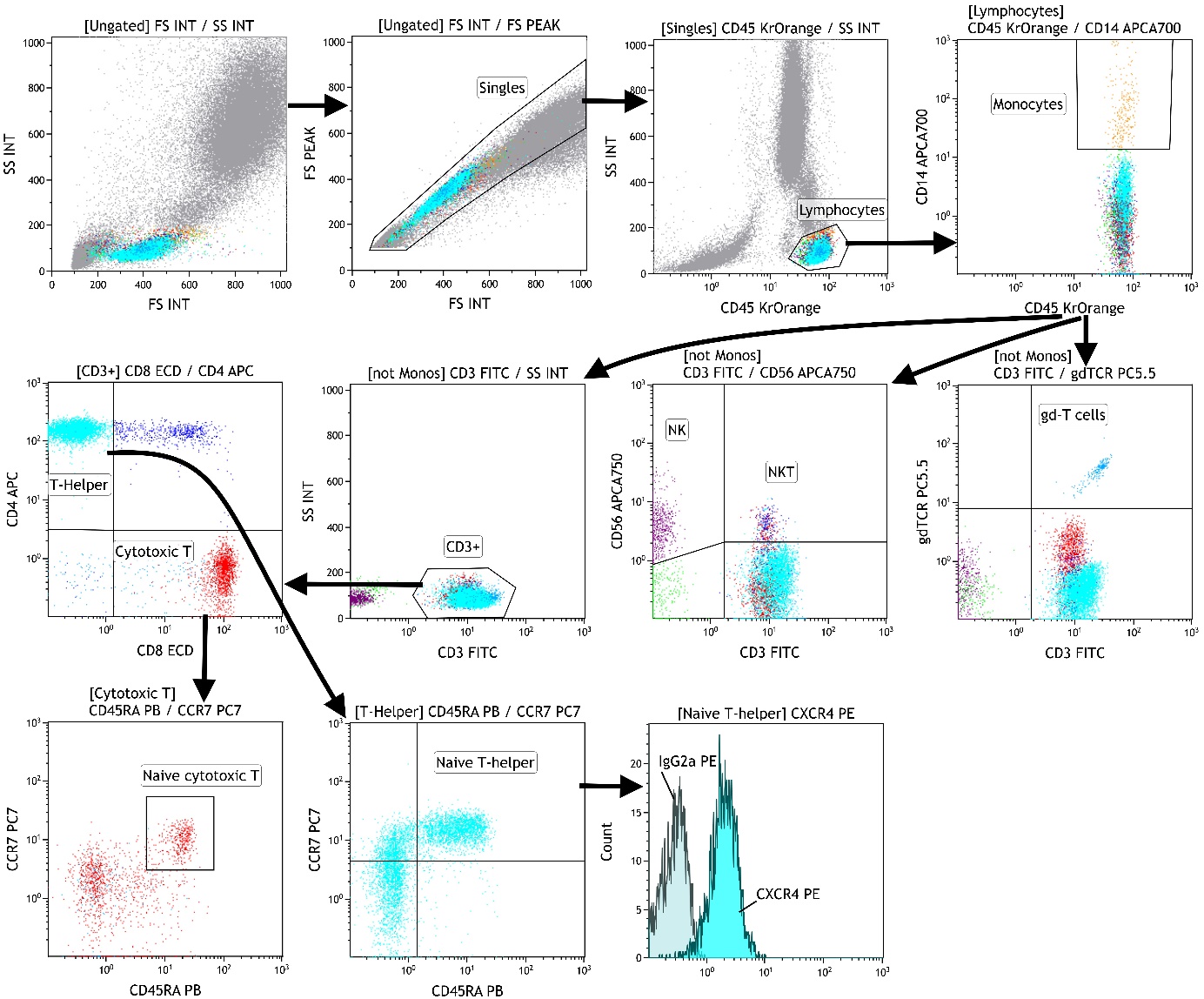
**

**A**

**B**

**
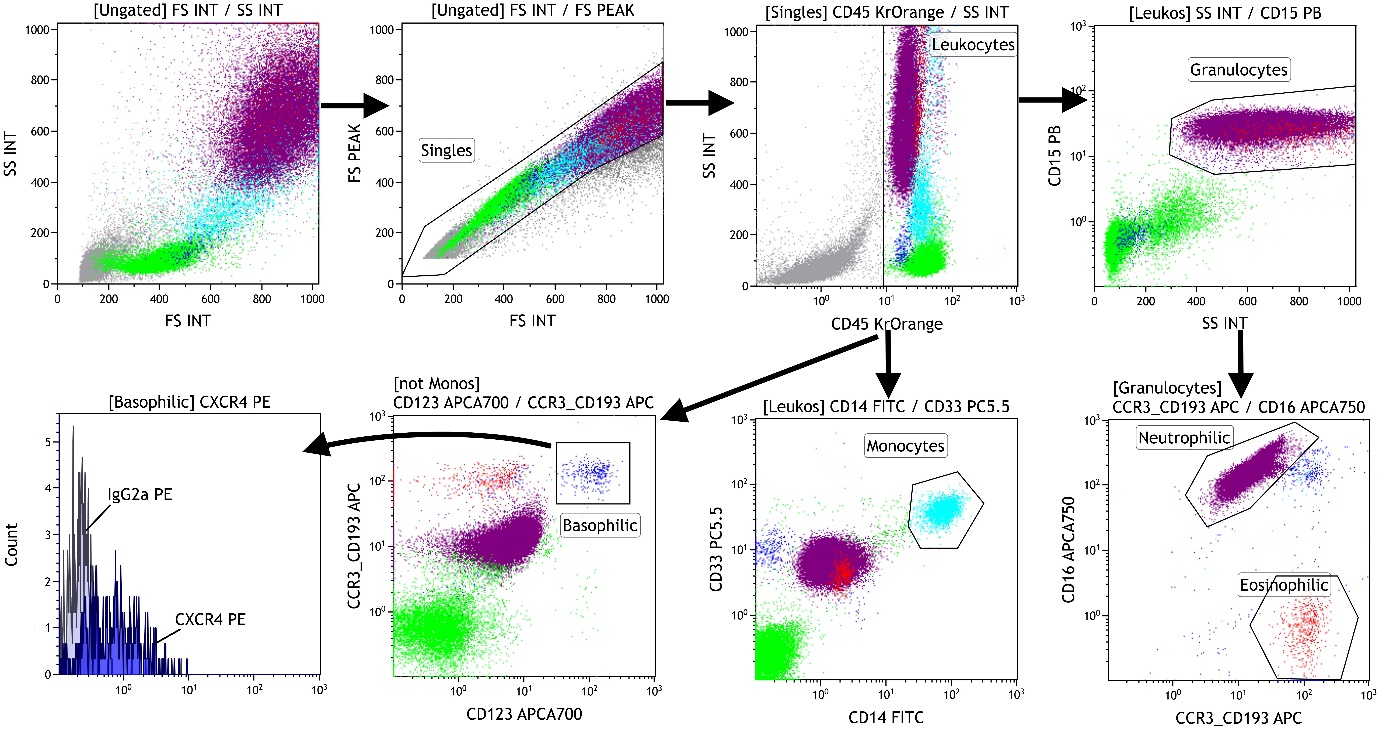
**


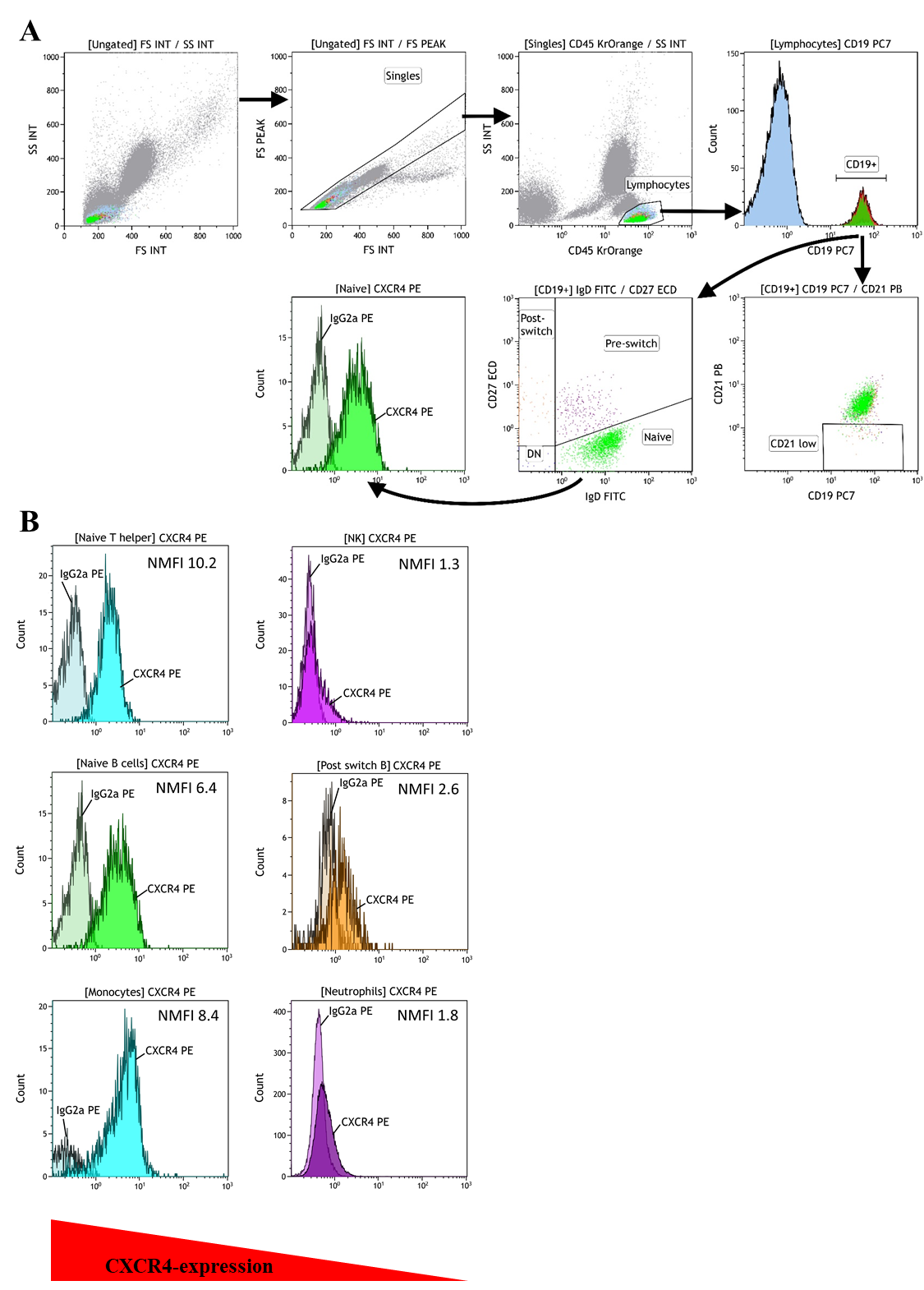


**C**

**Figure S1:** Flow cytometry of peripheral-blood leucocytes. (A) Gating strategy of the T cell panel: Singles were identified by forward scatter intensity (FS INT) vs. forward scatter peak (FS PEAK). Within singles, lymphocytes were identified by side scatter intensity (SS INT) vs CD45 expression. Monocytes were identified by CD14 expression to exclude them from further gates. NK, NKT, γδ-T, and T cells (CD3+) were identified with the respective antibodies. Within the T cells, T-helper and cytotoxic T cells were identified based on CD4 or CD8 expression. Naïve T-helper cells were identified within the T-helper compartment by CCR7- and CD45RA-positivity. Naïve cytotoxic T cells were identified by CCR7- and CD45RA-positivity within the cytotoxic T cells. Right blot, lower row: Representative blot of CXCR4-Expression on naïve T-helper cells (CXCR4 PE) compared to isotype control (IgG2a PE). (B) Gating strategy of the Myeloid panel: Singles were identified by forward scatter intensity (FS INT) vs. forward scatter peak (FS PEAK). Within singles, leukocytes were identified by side scatter intensity (SS INT) vs CD45 expression. Within leukocytes, granulocytes were detected by SS INT vs CD15 to define neutrophilic and eosinophilic granulocytes. Monocytes were identified by CD14 and CD33 expression. Basophilic granulocytes were identified by CCR3- and CD123-positivity. Left blot, lower row: Representative blot of CXCR4-Expression on basophilic granulocytes (CXCR4 PE) compared to isotype control (IgG2a PE). (C) (A) Gating strategy of the B cell panel: Singles were identified by forward scatter intensity (FS INT) vs. forward scatter peak (FS PEAK). Within singles, lymphocytes were identified by side scatter intensity (SS INT) vs CD45 expression. Within the lymphocyte gate B cells were defined as CD19 positive. Within the B cell gate (CD19+), five subpopulations were identified, based on CD21, CD27 or IgD expression. Left blot, lower row: Representative blot of CXCR4-Expression on naïve B cells (CXCR4 PE) compared to isotype control (IgG2a PE).

**
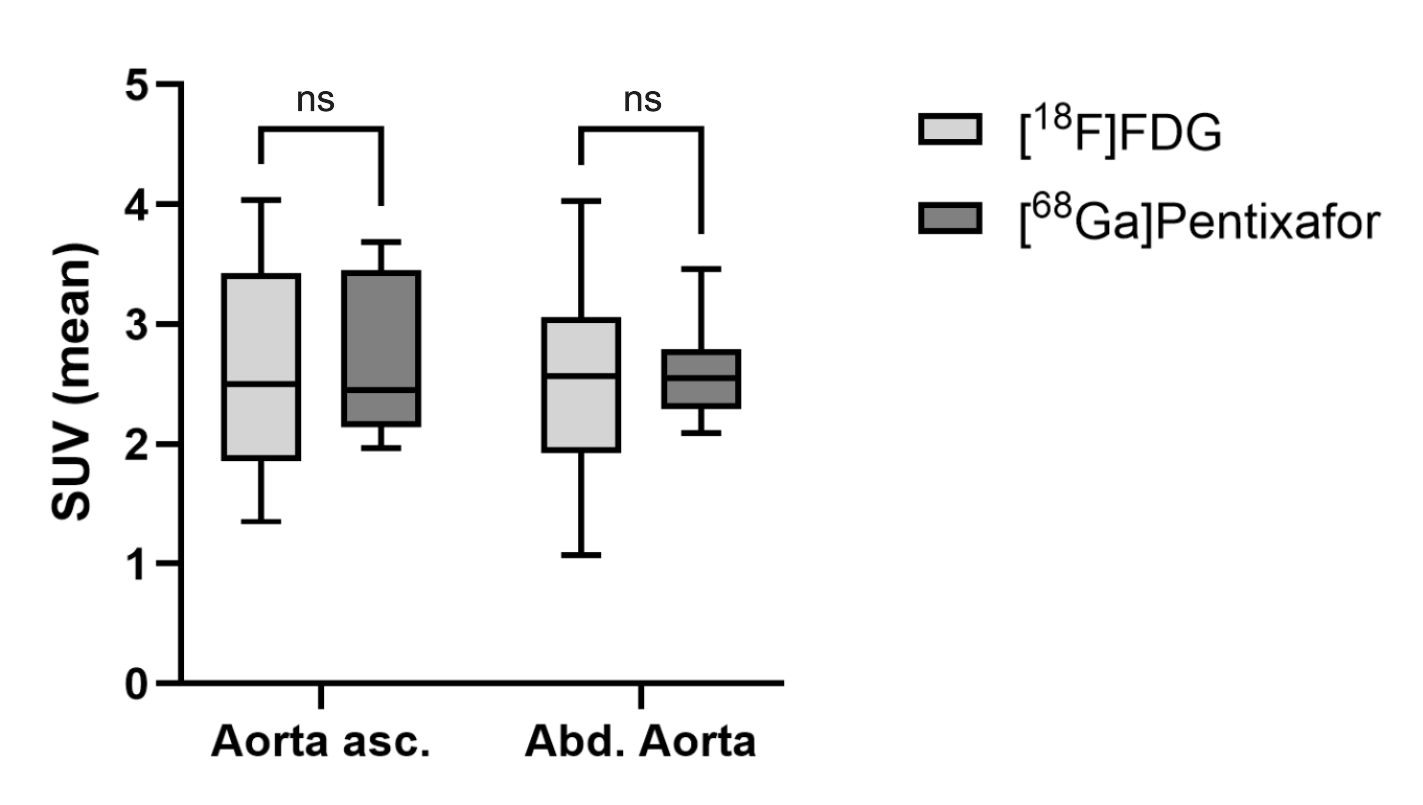
**

**Figure S2:** Blood pool activity in the ascending and abdominal aorta across tracers. Bar plots depict the mean standardized uptake values (SUVmean) for [^18^F]FDG and [^68^Ga]PentixaFor in the ascending (left) and abdominal aorta (right) across all GCA patients (n = 10). These data suggest that CXCR4 expression on circulating leukocytes does not lead to a relevant increase in unspecific blood pool activity at 1 h post-injection (ns = not significant).

| **Panel** | **Fluorochrome** | | | | | | | | | |
| --- | --- | --- | --- | --- | --- | --- | --- | --- | --- | --- |
|  | KrOrange | PC7 | PE | FITC | PC5.5 | PB | ECD | APC | APCA700 | APCA750 |
| Myeloid | CD45 | - | CXCR4 or IgG2a (isotype control) | CD14 | CD33 | CD15 | CD117 | CCR3 (CD193)^§^ | CD123 | CD16 |
| B cell | CD45 | CD19 | CXCR4 or IgG2a (isotype control) | IgD^*^ | - | CD21^†^ | CD27 | - | CD38 | - |
| T cell | CD45 | CCR7 | CXCR4 or IgG2a (isotype control) | CD3 | γδTCR | CD45RA | CD8 | CD4 | CD14 | CD56 |

**Table S1:** Antibodies for flow cytometry. Commercial availability of the fluorochrome-conjugated antibodies: All Beckman Coulter (Krefeld, Germany), except otherwise indicated: ^§^Invitrogen (Carlsbad, CA), ^*^BD Biosciences (San Jose, CA), ^†^Exbio (Prague, Czech Republic).

| **Panel** | **Leukocyte subpopulations** |
| --- | --- |
| Myeloid | Neutrophilic granulocytes: CD45^+^/CD15^+^/CD16^+^/CCR3^-^  Eosinophilic granulocytes: CD45^+^/CD15^+^/CD16^-^/CCR3^+^  Basophilic granulocytes: CD45^+^/CD14^-^/CD123^+^/CCR3^+^  Monocytes: CD45^+^/CD33^+^/CD14^+^ |
| B cell | Total B cells: CD45^+^/CD19^+^  Naïve B cells: CD45^+^/CD19^+^/CD27^-^/IgD^+^  Pre-switch memory B cells: CD45^+^/CD19^+^/CD27^+^/IgD^+^  Post-switch memory B cells: CD45^+^/CD19^+^/CD27^+^/IgD^-^  Double negative B cells: CD45^+^/CD19^+^/CD27^-^/IgD^-^  CD21low B cells: CD45^+^/CD19^+^/CD21^-^ |
| T cell | Total T cells: CD45^+^/CD14^-^/CD3^+^  T-helper cells: CD45^+^/CD14^-^/CD3^+^/CD4^+^  Cytotoxic T cells: CD45^+^/CD14^-^/CD3^+^/CD8^+^  γδ-T cells: CD45^+^/CD14^-^/CD3^+^/γδ-TCR^+^  Naïve T-cells: CD45^+^/CD14^-^/CD3^+^/CCR7^+^/CD45RA^+^  Naïve T-helper T cells: CD45^+^/CD14^-^/CD3^+^/CD4^+^/CCR7^+^/CD45RA^+^  Naïve cytotoxic T cells: CD45^+^/CD14^-^/CD3^+^/CD8^+^/CCR7^+^/CD45RA^+^  Natural killer cells: CD45^+^/CD14^-^/CD3^-^/CD56^+^  Natural killer T cells: CD45^+^/CD14^-^/CD3^+^/CD56^+^ |

**Table S2:** Characterization of the examined leukocyte populations by flow cytometry.


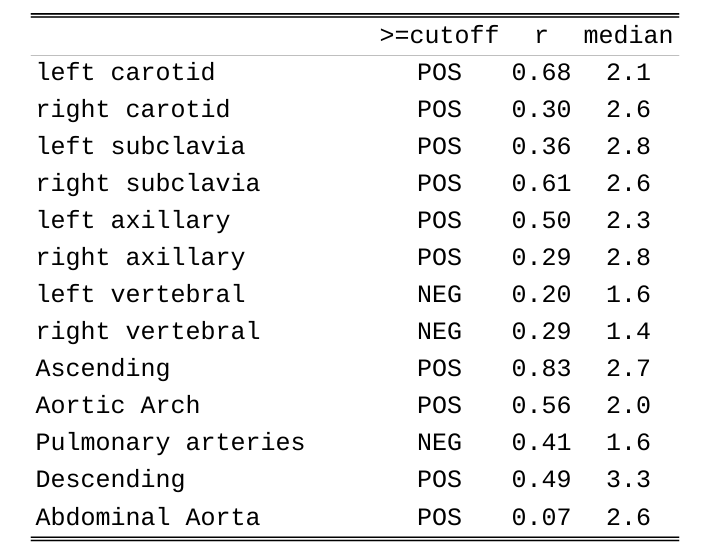


**Table S3:** Pearson correlations [^18^F]FDG-[^68^Ga]PEN per segment and exemplary classification of segments at [^18^F]FDG cutoff 2.

| **Vessel segment** | **TBR [^68^Ga]PentixaFor** | **TBR [^18^F]FDG** | **P-value** | **IQR [^68^Ga]PentixaFor** | **IQR [^18^F]FDG** |
| --- | --- | --- | --- | --- | --- |
| Average all vessel segments | 1.76 ± 0.76 | 2.43 ± 0.90 | 0.07 | 0.43 | 1.10 |
| Left carotid artery | 1.58 ± 0.7 | 2.19 ± 1.16 | 0.07 | 0.87 | 2.20 |
| Right carotid artery | 2.02 ± 1.45 | 2.62 ± 1.56 | 0.13 | 1.07 | 1.19 |
| Left subclavia artery | 1.75± 0.72 | 2.53 ± 1.39 | 0.1 | 1.02 | 2.57 |
| Right subclavia artery | 1.64 ± 0.56 | 2.72 ± 1.71 | 0.03 | 0.70 | 1.60 |
| Left axillary artery | 1.52 ± 0.75 | 2.49 ± 1.62 | 0.08 | 1.16 | 2.74 |
| Right axillary artery | 1.56 ± 0.65 | 2.89 ± 1.96 | 0.07 | 0.99 | 2.88 |
| Left vertebral artery | 1.39 ± 0.52 | 1.96 ± 1.25 | 0.08 | 0.52 | 0.45 |
| Right vertebral artery | 1.32 ± 0.5 | 1.59 ± 0.57 | 0.19 | 0.51 | 0.51 |
| Ascending Aorta | 1.93 ± 0.91 | 2.56 ± 0.9 | 0.01 | 0.23 | 1.03 |
| Aortic Arch | 1.91 ± 1.1 | 2.31 ± 0.9 | 0.24 | 0.45 | 1.34 |
| Pulmonary arteries | 1.92 ± 1.1 | 1.64 ± 0.24 | 1 | 0.52 | 0.34 |
| Descending Aorta | 2.13 ± 1.06 | 3.28 ± 1.63 | 0.04 | 0.72 | 1.17 |
| Abdominal Aorta | 2.24 ± 1.13 | 2.75 ± 1.17 | 0.26 | 0.41 | 0.74 |

**Table S4:** Target-to-background ratios (TBR) and interquartile range (IQR) of average and individual vessel segments of [^68^Ga]PentixaFor in direct comparison with [^18^F]FDG. Parameters are given in mean ± standard deviation.
